# The p53-Reactivating Small Molecule RITA Induces Senescence in Head and Neck Cancer Cells

**DOI:** 10.1371/journal.pone.0104821

**Published:** 2014-08-13

**Authors:** Hui-Ching Chuang, Liang Peng Yang, Alison L. Fitzgerald, Abdullah Osman, Sang Hyeok Woo, Jeffrey N. Myers, Heath D. Skinner

**Affiliations:** 1 Department of Head and Neck Surgery, The University of Texas MD Anderson Cancer Center, Houston, Texas, United States of America; 2 Department of Otolaryngology, Kaohsiung Chang Gung Memorial Hospital and Chang Gung University College of Medicine, Kaohsiung, Taiwan; 3 Department of Radiation Oncology, The University of Texas MD Anderson Cancer Center, Houston, Texas, United States of America; 4 Graduate School of Biomedical Sciences, The University of Texas MD Anderson Cancer Center, Houston, Texas, United States of America; Columbia University Medical Center, United States of America

## Abstract

TP53 is the most commonly mutated gene in head and neck cancer (HNSCC), with mutations being associated with resistance to conventional therapy. Restoring normal p53 function has previously been investigated via the use of RITA (reactivation of p53 and induction of tumor cell apoptosis), a small molecule that induces a conformational change in p53, leading to activation of its downstream targets. In the current study we found that RITA indeed exerts significant effects in HNSCC cells. However, in this model, we found that a significant outcome of RITA treatment was accelerated senescence. RITA-induced senescence in a variety of p53 backgrounds, including p53 null cells. Also, inhibition of p53 expression did not appear to significantly inhibit RITA-induced senescence. Thus, this phenomenon appears to be partially p53-independent. Additionally, RITA-induced senescence appears to be partially mediated by activation of the DNA damage response and SIRT1 (Silent information regulator T1) inhibition, with a synergistic effect seen by combining either ionizing radiation or SIRT1 inhibition with RITA treatment. These data point toward a novel mechanism of RITA function as well as hint to its possible therapeutic benefit in HNSCC.

## Introduction

Mutations in *TP53* are a common genetic alteration present in many types of solid tumor, including head and neck squamous cell carcinoma (HNSCC) [1], [2]. Our group and others have shown that *TP53* mutations are associated with increased resistance to radiation and chemotherapy in HNSCC cell lines *in vitro* and with poor outcomes in patients with HNSCC [3]–[5]. Unfortunately, therapeutic strategies to re-introduce wild-type (wt) p53 into tumors have been logistically challenging [6], and thus strategies are being explored for therapeutic re-activation of endogenous p53 instead [7]–[10]. One compound of interest, RITA (reactivation of p53 and induction of tumor cell apoptosis), is a small molecule that binds to the N-terminus of the p53 protein and induces a conformational change that can lead to restoration of normal p53 function [11], [12]. RITA can activate p53 downstream targets in both p53 wt [13], [14] and p53 mutant (mt) cells [15] in a variety of models.

RITA is thought to act primarily via the induction of apoptosis, and indeed RITA, alone or in combination with cisplatin, can induce apoptosis in many HNSCC cell lines [16], [17]. However, this effect is not universal. Cell lines that express wt p53 but do not undergo apoptosis in response to RITA treatment include the HNSCC cell line JHU-028 [17], the human osteosarcoma cell line SJSA and the human colon carcinoma cell line RKO [18].

Apoptosis is not the only cellular fate after p53 activation. Numerous studies have found that activation of p53 in response to a variety of stimuli in cancer cells leads to accelerated senescence [7]. Although the final outcome of the cell after it enters senescence is unclear, several studies have linked the induction of senescence with response to therapeutic agents. We have observed that radiation [3] and cisplatin [4] inhibited cell growth by inducing senescence in wt p53 HNSCC cells. Consistent with this observation, HNSCC cells expressing mt p53 were found to be resistant to radiation or cisplatin, largely because of the lack of a senescence response. We further observed that many of these same cell lines are also resistant to therapy-induced apoptosis [3], [4]. Thus, at least in this model, induction of senescence seems to reflect a favorable treatment outcome.

The aim of this study was to determine the effect of RITA on survival, proliferation, and induction of senescence in several human HNSCC cell lines. We further sought to understand the mechanisms by which these effects occur.

## Materials and Methods

### Cell lines

The HNSCC cell lines used in this study were generous gifts from Dr. Jeffrey Myers (The University of Texas MD Anderson Cancer Center and have been previously characterized [19]. HN30 and HN31 cell lines were derived from a primary tumor and lymph nodal metastasis of pharyngeal squamous cell carcinoma respectively. The whole exome of these two cell lines has been sequenced for a separate project and, with the exception of TP53, no other discordant mutations between the two cell lines were observed. The PCI-13 cell line was derived from an oral cavity squamous cell carcinoma. All cell lines were maintained in Dulbecco modified Eagle medium containing 10% fetal bovine serum, penicillin/streptomycin, glutamine, sodium pyruvate, nonessential amino acids and vitamins. Techniques for stably knocking down p53 in HN30 cells (which have wt p53) and HN31 cells (which have mt p53) are described elsewhere [3]. PCI-13 cells, which have no endogenous p53, were engineered to express *TP53* overexpression constructs (wt p53, A161S, G245D), which were generated and inserted into a pBabe retroviral vector containing a puromycin-resistance insertion (pBaBe-puro; Addgene) by using standard cloning techniques. HN31 cells were transfected with short-hairpin RNA (shRNA) specific for SIRT-1 (Silent information regulator T1) or control scrambled shRNA via lentiviral vectors containing the puromycin-resistance gene from Santa Cruz Biotechnology (Santa Cruz, CA), according to the manufacturer's instructions. Lentiviral-transfected control cells (HN31-C2) and their shRNA, stable SIRT-1-knockdown counterpart (HN31-S19) were isolated by immunoblotting after clones were screened. β-actin was used as an internal loading control.

### Antibodies and immunoblotting

Protein and phosphorylated protein expression levels were assessed by immunoblotting of whole-cell lysates from cells treated or not treated with RITA. The following primary antibodies were used: p53 (DO-1) and phospho-serine 15 p53 from Santa Cruz Biotechnology; p21 from Calbiochem; p-p53(Ser-15), p53-upregulated modulator of apoptosis (PUMA), murine double minute2 (MDM2), checkpoint kinase 2 (Chk2), phosphorylated Chk2 (p-Chk2, Thr68), SIRT1, and β-actin from Cell Signaling Technology (Danvers, MA). Goat anti-mouse and anti-rabbit secondary antibodies conjugated to horseradish peroxidase were purchased from Cell Signaling and Santa Cruz Biotechnology, respectively.

### Reagents

RITA was purchased from Cayman Chemical (Ann Arbor, MI) and the protein kinase inhibitor staurosporine was purchased from Sigma (St Louis, MO). The SIRT1 inhibitor Tenovin-6 was obtained from Santa Cruz Biotechnology (Dallas, TX). RITA was dissolved in 100% dimethylsulfoxide (DMSO) to a stock concentration of 50 mM and stored as aliquots until use. Staurosporine and Tenovin-6 were dissolved in 100% DMSO and water, respectively, to stock concentrations of 1 M and stored as aliquots until use.

### Growth suppression assays

The cell viability assay has been described previously [20]. Briefly, 2000 cells/well were plated in 96-well plates, treated with various concentrations of RITA for up to 72 hours, and viability was assessed with an MTT assay. For the colony-formation assay [3], HNSCC cells were seeded in 6-well plates for 24 hours, treated with RITA, and cultured for 10–14 days. Cells were fixed in a 3% crystal violet/10% formalin solution and colonies containing more than 50 cells were counted with ImageJ software.

### Senescence-associated- β-galactosidase staining

Senescence-associated β-galactosidase (SA-β-gal) staining was carried out according to the manufacturer's instructions (Cell Signaling Technology). Briefly, HNSCC cells were plated in 6-well plates and treated with RITA at various concentrations for various times (typically 5 days), after which cells were fixed for 10 minutes and stained for SA-β-gal activity overnight at 37°C. Flattened and blue-staining cells were scored as senescent and reported as a percentage of all cells observed per high-power field.

### Statistical analyses

Data were pooled for analysis from several independent experiments, and cell-based assays were done in triplicate. Two-tailed Student's *t* tests were used to assess differences in senescence and for other unpaired group comparisons. For all comparisons, *P*<0.05 was considered statistically significant.

## Results

### Effects of RITA on p53 and p53-associated proteins

**Figure 1:**
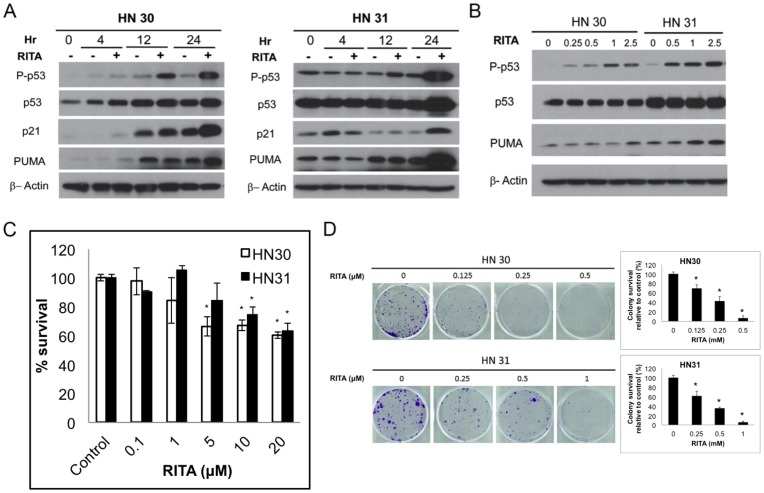
Effects of RITA on p53 signaling in head and neck cancer cells. (A and B) HN30 (wild-type p53) and HN31 (mutated p53) head and neck cancer cells were treated with RITA for the indicated times (A) and doses (B) and the expression of p53 and its targets were evaluated by immunoblotting. (C) Cells were treated with RITA (0.1 µM–20 µM) for 72 hours and assessed via MTT assay. Data are expressed as means ± S.E from three experiments. (D) HN30 and HN31 cells were treated with RITA at the indicated doses for 10–14 days, after which colonies were fixed, stained, and quantified. Representative images from three experiments with similar results are shown. Quantitative data are expressed as means ± S.E from three experiments. * - indicates p<0.05 versus untreated control.

We used a pair of isogenic HNSCC cell lines, one with wt p53 (HN30) and the other with mt p53 (HN31), to test the effect of RITA on the expression and phosphorylation of p53 and other related proteins. RITA strongly induced the phosphorylation of p53 and p21 after 12 hours in HN30 cells and after 24 hours in HN31 cells (Fig. 1A). PUMA protein levels were also elevated after 24 hours of exposure to RITA in both cell lines. The increases in p53 expression, p53 phosphorylation, and PUMA expression at 24 hours were all dose-dependent (Fig. 1B). These results indicate that RITA can activate downstream targets of p53 signaling in these isogenic cell lines with wt or mt p53.

### RITA inhibits growth of head and neck cancer cell lines

Several studies have that RITA can induce tumor cell death [15], [18]. Initially, we tested whether RITA can suppress the growth of HN30 and HN31 cells. Using a short-term cell proliferation assay, we found that RITA, at concentrations over 5 µM, induced modest growth suppression in both HN30 and HN31 cells after 72 hours of continuous treatment (Fig. 1C). In a longer-term colony formation assay, RITA was found to significantly reduce the number of colonies formed by both cell lines at all doses tested (p<0.05) (Fig. 1D).

### RITA induces senescence in head and neck cancer cell lines

**Figure 2:**
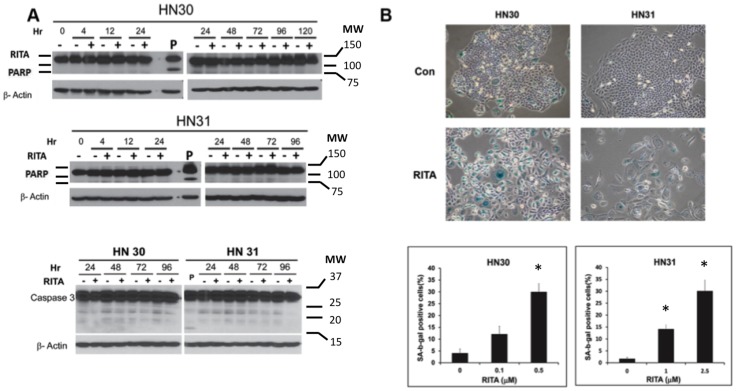
Effects of RITA on proliferation of head and neck cancer cells. (A) PARP and caspase 3 cleavage were examined via western blotting after exposure of cells to RITA at 1 µM for the indicated periods. P, staurosporine (1 µM for 8 hours) was used as a positive control. (B) HN30 and HN31 cells seeded in 6-well tissue culture plates were treated with the indicated concentrations of RITA for 5 days, after which cells were fixed and stained for senescence-associated -β-galactosidase (SA-β-gal) activity. Representative images from three experiments with similar results are shown. In each treated or untreated well, five random field selections were made and the number of cells with senescent morphology and with blue staining were counted under 20X magnification (Olympus IX71). * - indicates p<0.05 versus untreated control.

We previously showed that the dominant mode of response to either physiologically relevant concentrations of cisplatin [4] or standard doses of radiation [3] in p53 wild type HNSCC cell lines is not apoptosis. Similarly, in the current model, minimal caspase and PARP cleavage was observed following exposure to RITA (Fig. 2A). Thus, to test the hypothesis that the observed anti-proliferative response of HN30 and HN31 cells to RITA was at least partially due to the induction of senescence, we analyzed SA-β-gal activity, a marker of cellular senescence. We found that RITA affected cell morphology and significantly increased SA-β-gal staining in a dose-dependent manner in both cell lines (p<0.05; Fig. 2B), indicating that RITA treatment led to accelerated senescence in these isogenic cell lines.

### p53 status and RITA-mediated growth inhibition in HNSCC cell lines

**Figure 3:**
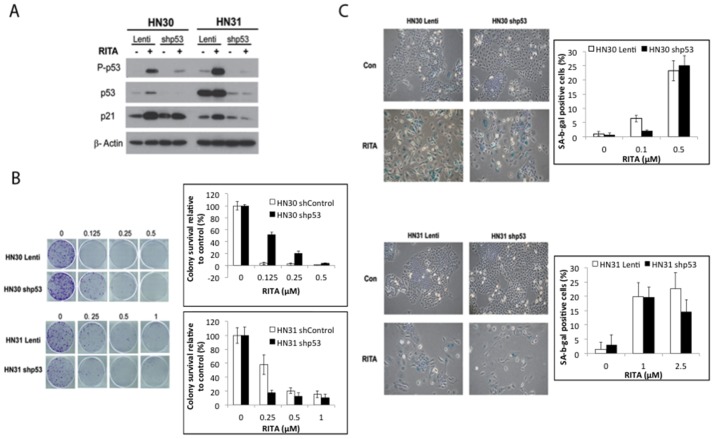
Effects of shRNA mediated p53 inhibition on RITA response in HN30 and HN31 cells. (A) Levels of total and phosphorylated p53 and p21 were measured in HN30 and HN31 lentiviral-transfected control cells and their short-hairpin RNA, p53-stable-knockdown counterparts after treatment with 1 µM RITA for 72 hours. β-actin was used as an internal loading control. (B) HN30 and HN31 lentiviral-transfected control cells and their p53-stable-knockdown counterparts were treated with RITA at the indicated doses for 10–14 days, after which colonies were fixed, stained, and quantified. Representative images from three experiments with similar results are shown. Quantitative data are expressed as means ± S.E from three experiments. In all cell lines (control and sh53), RITA treatment led to significantly (p<0.05) decreased colony formation at all doses tested. (C) HN30 and HN31 lentiviral-transfected control cells and their p53-stable-knockdown counterparts were seeded in 6-well tissue culture plates and treated with the indicated concentrations of RITA for 5 days, after which cells were fixed and stained for senescence-associated -β-galactosidase (SA-β-gal). Representative images from three experiments with similar results are shown. With the exception of 0.1 µM in HN30-shp53, all doses of RITA in all cell lines led to significantly (p<0.05) increased SA-β-gal compared to untreated control. No significant differences were observed between control and shp53 in either cell line.

Next, to investigate whether RITA-induced senescence depends on p53 status, we treated the stable-p53-knockdown cell lines HN30-shp53 and HN31-shp53 with RITA and found that knockdown of p53 protein expression markedly reduced both the expression and phosphorylation of p53 (Fig. 3). However, baseline p21 expression was unchanged in the HN30 (wt p53) cells (Fig. 3A). We further found that p53 inhibition had only a partial rescue effect on RITA-induced growth inhibition and colony formation in HN30 cells, and it had no significant rescue effect on HN31 cells (Fig. 3B). Moreover, p53 knockdown did not affect RITA-induced senescence in either type of p53-knockdown cells (Fig. 3C).

**Figure 4:**
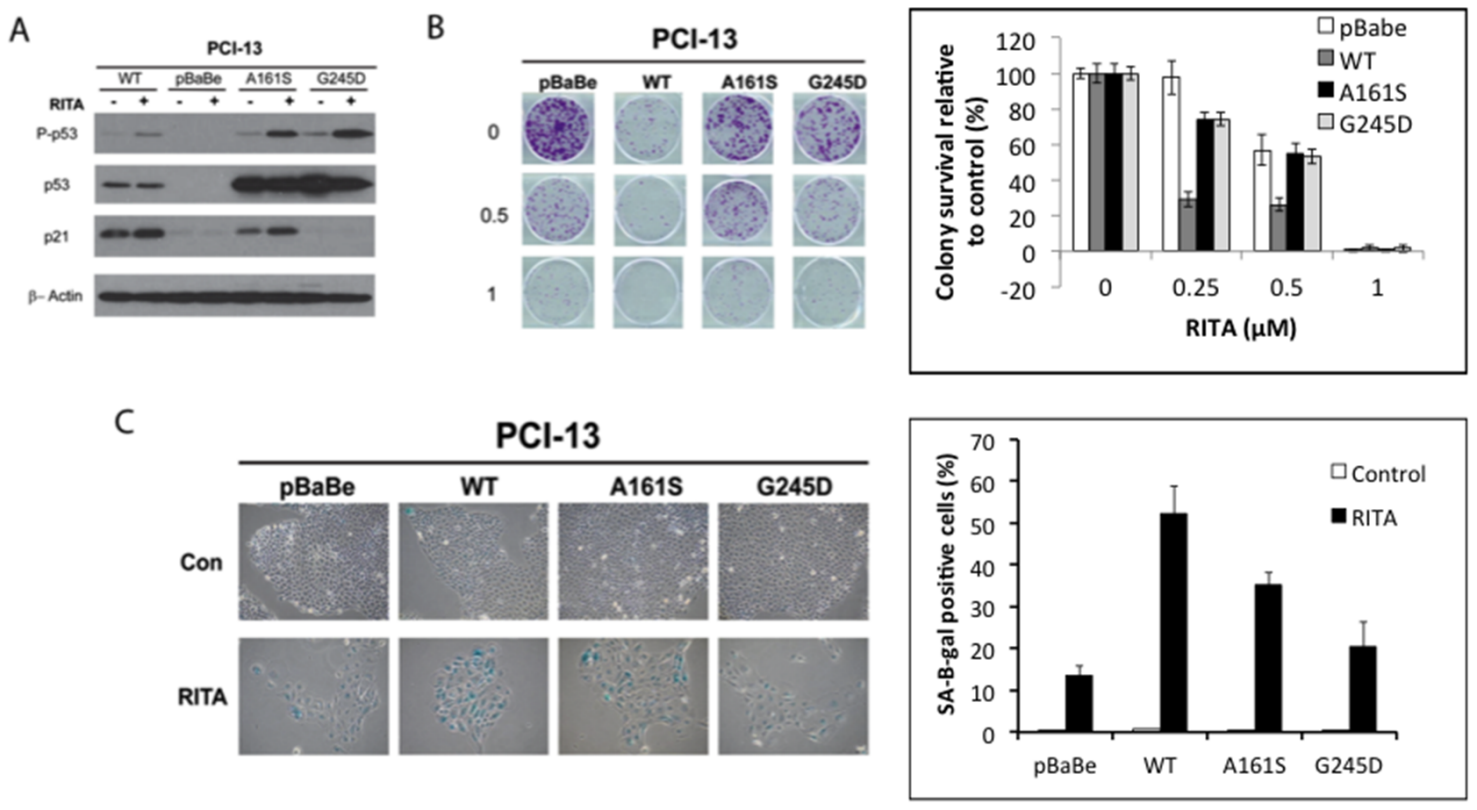
Effects of RITA in PCI-13 (p53 null) cells expressing mutant p53. (A) PCI-13 (p53 null) cells expressing vector control or the indicated p53 constructs were treated with RITA 2.5 µM for 72 hours after which protein lysates were prepared and levels of total and phosphorylated p53 and p21 were assessed by western blotting. β-actin was used as an internal loading control. (B) PCI-13 cells expressing the indicated constructs were seeded on 6-well plates, treated with RITA at the indicated concentrations, and counted in a clonogenic assay. With the exception of pBabe cells treated at 0.25 µM, all cell lines at all tested concentrations of RITA exhibited significantly decreased colony formation compared to untreated control (p<0.05). (C) PCI-13 cells expressing the indicated constructs were seeded in 6-well tissue culture plates and treated with 0.25 µM of RITA after which cells were fixed and stained for senescence-associated -β-galactosidase (SA-β-gal) activity. Representative images from three experiments with similar results are shown. All cell lines exhibited significantly increased SA-β-gal staining compared to untreated control (p<0.05).

Next, we tested whether reconstitution of wt p53 and mt p53 in p53-null PCI-13 cells would confer susceptibility to RITA. For these experiments, pBabe (vector control), wt p53, and the A161S and G245D mutant p53 constructs were re-introduced into PCI-13 cells to produce the PCI-13-pBABE, PCI-13-WT, PCI-13-A161S, and PCI-13-G245D cell lines. In contrast to p53-positive cells, p53 and phosphorylated p53 were not detected in the PCI-13 pBabe control cells (Fig. 4A). Also, p21 was expressed only by the wt expressing cells and by one of the mt p53-expressing cells (PCI-13-A161S).

A long-term colony formation assay (Fig. 4B) and SA-β-gal staining (Fig. 4C) showed that RITA significantly inhibited cell growth and induced senescence in all of the PCI-13 cell lines regardless of p53 status, including the p53-null parental line (p<0.05). Thus, the presence of the p53 protein does not seem to be required for RITA to exert its effects.

### RITA-induced DNA damage response in HNSCC cell lines is associated with SIRT1 downregulation

**Figure 5:**
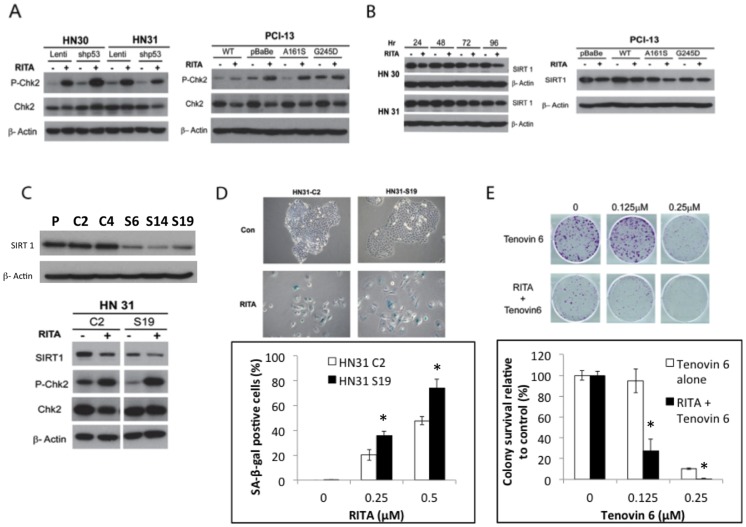
RITA leads to CHK2 phosphorylation and SIRT1 inhibition in head and neck cancer cell lines. (A) HN30 or HN31 cells transfected with lentiviral control or short-hairpin RNA, p53-stable-knockdown constructs were treated with 1 µM RITA 1 for 72 hours, after which protein lysates were prepared and levels of total and phosphorylated Chk2 assessed by immunoblotting. Right: PCI-13 cells transfected with the indicated constructs were treated with 2.5 µM RITA for various periods, after which protein lysates were prepared and levels of total and phosphorylated Chk2 were assessed. (B) HN30, HN31, and PCI-13 cells with the indicated p53 constructs were treated with RITA for the indicated periods, and lysates were assessed for SIRT1 protein expression by western blotting. β-actin was used as an internal loading control. (C) HN31 cells stably transduced with lentiviral control vectors (C) or SIRT1 shRNA (S) were initially assayed for inhibition of SIRT-1 expression. Representative clones were then treated with 2.5 µM RITA for 72 hours, after which the indicated proteins were extracted and analyzed by western blotting. (D) HN31 cells stably transduced with lentiviral control vectors (C2) or SIRT1 shRNA (S19) were seeded in 6-well tissue culture plates and treated with the indicated concentrations of RITA for 5 days, after which cells were fixed and stained for senescence-associated -β-galactosidase (SA-β-gal) activity. Representative images from three experiments with similar results are shown. * - indicates significantly increased compared to control vector at the indicated dose (p<0.05). (E) HN31 cells were seeded on 6-well plates and treated with RITA (0.25 M) with or without Tenovin 6 (0.125 or 0.25 µM) for 10 days, after which colonies were fixed, stained, and quantified. * - indicates significantly decreased compared to Tenovin 6 alone group at the indicated dose. Data are normalized to either vehicle-only or RITA treatment conditions. Representative images from three experiments with similar results are shown. Data are expressed as means ± S.E from three experiments.

RITA is also known to induce the DNA damage response [21], [22]. Checkpoint kinase 2 (Chk2) is an important downstream target of the DNA damage response and leads to cell cycle arrest, apoptosis, or senescence. Notably, activated Chk2 can induce p53-independent senescence in cancer cells [23], [24]. These results, with our finding that RITA inhibits HNSCC cell growth and can induce senescence even in the absence of p53, led us to investigate the effect of RITA on Chk2 expression and phosphorylation status. We found that Chk2 protein was phosphorylated at its activation site, Thr68, after exposure to RITA in HN30, HN31 and PCI-13 cells, regardless of p53 status (Fig. 5A).

Next, because Chk2 seems to activate cellular senescence by inhibiting SIRT1 [25], and because knockdown or inhibition of SIRT1 activity also can induce senescence-like growth arrest in the absence of functional p53 [26]–[28], we evaluated SIRT1 expression and found that RITA led to decreased SIRT1 expression in all cell lines evaluated, regardless of p53 status (Fig. 5B).

To test this effect further, we assessed whether depletion of SIRT1 by shRNA would enhance the biological effect of RITA. We found that shRNA-mediated inhibition of SIRT1 decreased SIRT1 protein levels and increased phosphorylated Chk2 levels after exposure to RITA (Fig. 5C). Moreover, RITA treatment substantially enhanced SA-β-gal staining in HN31-S19 (SIRT1-knockdown) cells compared with HN31-C2 (control-transfected) cells (Fig. 5D). Finally, the combination of RITA and the SIRT1 inhibitor Tenovin 6 produced a synergistic cell growth inhibition effect (Fig. 5E). Collectively, these results suggest that RITA can inhibit SIRT1 expression and that this effect contributes to RITA-induced senescence.

### RITA increases radiosensitivity of mutant p53-expression HNSCC

**Figure 6:**
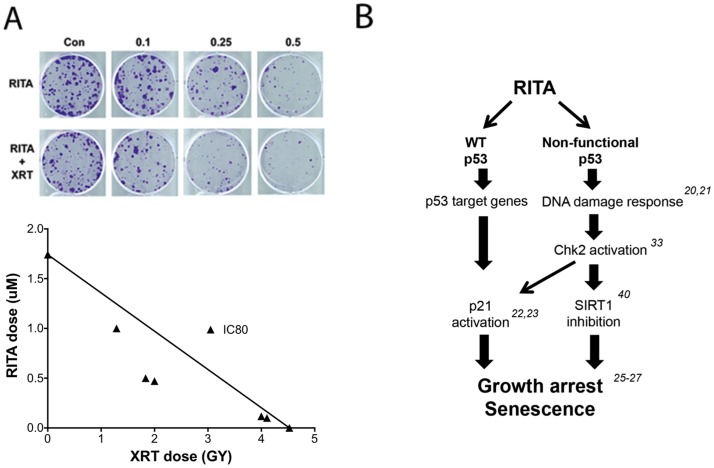
The effect of RITA and radiation therapy combination on colony formation. (A) HN31 cells were plated at 1000 cells/well in 6-well plates, treated with RITA for 24 hours, and treated with 2 Gy of radiation. Numbers of colonies were counted and used to calculate the surviving fraction. The results are presented as a standard isobologram, with the solid line representing an additive effect and the points representing the dose resulting in 80% inhibition (IC80) (B) Proposed mechanism of action of RITA in the context of different p53 mutation status.

On the basis of our previous finding that radiosensitivity in HNSCC cells is strongly related to their ability to undergo senescence after irradiation, specifically that HN31 mt p53 cells are radioresistant and have low levels of radiation-induced senescence, and our current findings that RITA decreased viability and increased senescence in HN31 cells, we investigated whether RITA could act as a radiosensitizer. We found that pretreatment of HN31 cells with RITA for 24 hours followed by irradiation resulted in synergistic inhibition of cell growth, when comparing the IC80 (Fig. 6A). Specifically, the mutually non-exclusive combinatorial index (CI) was calculated using the method of Chou & Talalay [29]. The combination of RITA and radiation resulted in a CI of 0.50, with a CI of less than 1 indicating synergy.

## Discussion

We found in the present study that RITA could inhibit growth and induce senescence in HNSCC cells, even in the absence of p53 or in the context of depletion of p53 expression, and that this phenomenon was associated with increased Chk2 activation and depends at least in part on SIRT1. These findings stand in contrast to previous studies showing that RITA functions primarily via the induction of apoptosis via activation of p53 signaling; rather, our findings indicate that RITA can lead to senescence in HNSCC cells among other effects and is not entirely dependent on p53 expression.

Although many of the pathways involved in senescence are mediated by p53, particularly in the context of p53 phosphorylation, senescence can also occur in the absence of this protein, suggesting that p53-independent mechanisms can also mediate therapy-induced senescence of tumor cells [30]–[34]. For example, doxorubicin was shown to induce a senescent phenotype in the p53-null Saos-2 cells, in SW480 and U251 cells that express mutant p53, and in HeLa and Hep-2 cell lines, in which p53 function has been inhibited [35].

Other groups have reported that RITA can not only induce p53 but also induce a concurrent DNA damage response [21], [22]. The DNA damage response involves two major signaling pathways, the sensor kinases ataxia telangiectasia mutated (ATM) and ataxia telangiectasia and Rad3-related (ATR). These kinases activate the downstream effector kinases Chk2 and Chk1. Chk2 can trigger replicative senescence via p53/p21 or other pathways in response to telomere dysfunction and DNA damage [36]. Recently, Chk2 was shown to modulate the cellular response to RITA [22]. Our findings that RITA treatment prompted an increase in Chk2 phosphorylation regardless of p53 status are consistent with this observation (Fig. 5).

Under conditions of cellular stress or DNA damage, Chk2 activation can lead to a reduction in SIRT1 expression and senescence [25]. SIRT1 is a highly conserved histone deacetylase that is known to mediate cellular metabolism, aging, and response to stress [37]. Overexpression of SIRT1 has been shown to inhibit cellular senescence in a variety of different malignancies [38]. In addition, SIRT1 is overexpressed in chemoresistant cells, and inhibiting SIRT1 can suppress tumor growth in some models [27], [38]–[40]. Indeed, in the current study we found that RITA inhibited SIRT1 expression in all cell lines tested, regardless of p53 status (Fig. 5B). Moreover, treatment with a SIRT1 inhibitor or SIRT1-specific shRNA led to decreases in growth and increases in senescence in conjunction with RITA treatment. Although inhibition of SIRT1 is thought to induce senescence primarily by interacting with p53, at least one group has shown that doxorubicin can induce senescence in SCC cells that lack p53 via inhibition of SIRT1 [26]. Additionally, one group has reported that inhibition of SIRT1 can induce senescence in H1299 (p53 null) via decreased activity in Ras/MAPK signaling. These observations may provide a link to the observed effects of RITA, even in HNSCC cells lacking p53 protein. On the basis of our findings from the current study, as well as the work of others, we propose that RITA induces growth arrest and senescence by at least two different pathways (Fig. 6B). In p53-competent cells, RITA likely acts primarily via canonical p53 targets, which is the dominant effect of the drug. The final outcome of this activation may be either apoptosis or senescence depending on the context. Conversely, a smaller, but still significant effect on cell viability is seen in p53 defective cell lines. However, in the absence of functional p53 or any p53 protein, RITA seems to exert at least some of its effects via activating the DNA damage response and inhibiting SIRT1 expression. The exact nature of this dual function will require further study.

We previously linked response to cisplatin and radiotherapy with the induction of senescence, showing that cells that are more resistant to these drugs are also resistant to senescence induction [3], [4]. Although the desirability of therapy-induced senescence as a response to clinically used therapies is a matter of significant debate [41], for cell types that are highly resistant to other forms of cell death or arrest, senescence may be an alternate therapeutic outcome. Our finding that low doses of RITA (0.1–0.5) could selectively sensitize highly resistant HNSCC cells to therapy in vitro [3] suggests that the addition of RITA may prove to be a viable strategy for sensitizing tumors to radiation, the most commonly used treatment for HNSCC.
